# The Effect of Bangerter Filters on Visual Acuity and Contrast Sensitivity With External Noise

**DOI:** 10.3389/fnins.2022.804576

**Published:** 2022-05-13

**Authors:** Pan Zhang, Hanlin Wang, Weicong Ren, Huanhuan Guo, Jiayi Yang, Jiayu Tao, Zhijie Yang, Ying Li, Lijun Chen, Yajing Zhang, Di Wu

**Affiliations:** ^1^Department of Psychology, Hebei Normal University, Shijiazhuang, China; ^2^Department of Psychology, Chengde Medical University, Chengde, China; ^3^Department of Psychiatry, Beijing Children’s Hospital, Capital Medical University, National Center for Children’s Health, Beijing, China; ^4^Department of Psychology, Shandong Normal University, Jinan, China; ^5^Department of Medical Psychology, Air Force Medical University, Xi’an, China

**Keywords:** Bangerter filter, visual acuity, contrast sensitivity, external noise, perceptual template

## Abstract

It is critical to address the relationship between density label of Bangerter filters and expected visual acuity, and how filters modulate contrast sensitivity (CS) at different spatial frequency and external noise levels. In the current study, the monocular visual acuity and CS at ten spatial frequencies and three noise levels were measured in normal subjects wearing no filters, 0.8, 0.4, or 0.2 Bangerter filters. Compared with the baseline condition (no filter worn), Bangerter filters degraded both visual acuity and contrast sensitivity function (CSF) in zero-noise conditions, but the reduction of both visual functions did not correlate with each other at any filter level. In addition, the stronger the filter was, the worse both visual functions became. In contrast, when external noise was present, filters improved the contrast sensitivity at low frequencies but deteriorated it at intermediate and high spatial frequencies. The perceptual template model was used to reveal the corresponding mechanism accounted for filter-induced visual function changes. Although the internal process in visual system should not be affected by the filters, the measurement of parameters was biased. To be specific, (1) the internal additive noise was elevated at all frequencies; (2) the perceptual template was improved at low spatial frequencies but impaired at intermediate spatial frequencies; and (3) the changes in both factors were highly dependent on filter intensity. We conclude that Bangerter filters influence visual acuity and contrast sensitivity differently and that their effect on contrast sensitivity depends on spatial frequency and noise.

## Introduction

Amblyopia is a developmental visual disorder and exhibits visual function loss, which cannot be explained by structural or pathological abnormalities (Daw, 1998). It is often related to anisometropia, strabismus, or cataracts and affects at 2–4% of population in western countries (Faghihi et al., 2017). The standard amblyopia treatment is to cover children’s fellow eye, which forces the visual cortex to process the visual input from the weaker (amblyopic) eye. Compared with traditional occlusion therapy, the approaches of blurring one eye recently received great attention due to their fewer compliance issues. Such methods include using atropine to dilate the pupil and paralyze accommodation (Medghalchi and Dalili, 2011) and attaching a Bangerter foil on the spectacle lens to diffuse one eye (Lang, 1999).

The current study focuses on the influence of Bangerter filters on visual function. As a popular approach in amblyopia treatment, Bangerter filters are used to reduce the visual acuity of normal eyes to certain levels, depending on the filters’ intensity. Without changing the phase spectra, Bangerter filters can attenuate the stimuli at intermediate to high spatial frequencies (Pérez et al., 2010). The efficiency of Bangerter filter treatment in amblyopic patients has been demonstrated to be comparable to that of traditional patching (Pediatric Eye Disease Investigator Group Writing Committee, Rutstein et al., 2010). In addition to amblyopic therapy, it is quite common to simulate the visual defects of several diseases, e.g., cataracts (Janknecht and Funk, 1995; Mauck et al., 1996) and vitreous haze in uveitis (Davis et al., 2010), by Bangerter filters.

Scientists identify the intensity of a Bangerter filter by checking its nominal strength. For instance, the 0.1 filter is supposed to attenuate visual acuity to 20/200. Thus, a worse visual acuity will be obtained when using a filter with a smaller number of label. Unfortunately, it has been reported that visual acuity reduction by Bangerter filters did not correspond closely to the labeled strength designation (Odell et al., 2008; Pérez et al., 2010; Li et al., 2012). For instance, Pérez et al. (2010) measured the influence of Bangerter foils (Ryser Optik, St. Gallen, Switzerland) on visual acuity and found that the visual acuity of subjects with a 0.8 filter was only 0.7. In Odell et al.’s (2008) study, the distance optotype acuity loss induced by 1.0, 0.8, and 0.4 filters was similar, whereas subsequent filters caused progressive degradation. However, Odell et al. (2008) used different manufacturers of Bangerter filters (Fresnel Prism and Lens Co., LLC, Eden Prairie, MN, United States). Li et al. (2012) found that the 0.8, 0.6, and 0.4 filters cause comparable degradation on letter acuity, but they did not report the name of Bangerter manufacturer. It is also important to note that only the subjects in Odell et al.’s (2018) study were not allowed to search for a less blurred part in the filter, while other studies did not make any statement. Since Bangerter filters made by Ryser are commonly used in China, our first aim is to evaluate the effect of Ryser Bangerter filters with different levels on visual acuity. At the same time, strict instruction was performed to exclude the possibility of viewing stimuli through a clear part of the filter.

On the other hand, unlike visual acuity, contrast sensitivity (CS) denotes subjects’ performance in distinguishing the luminance difference between adjacent areas (Campbell, 1983; Pelli and Bex, 2013). Contrast sensitivity is typically measured using sinusoidal grating patterns as targets and strongly depends on spatial frequencies. Contrast sensitivity can be used to predict visual performance with more complex visual material. For example, a previous study indicated that Air Force pilots’ contrast sensitivity, not their visual acuity, predicted the performance in a simulated air-to-ground target detection task (Ginsburg et al., 1983). Thus, how contrast sensitivity is modulated by a Bangerter filter is an interesting question. This issue has been partly examined by previous studies (Odell et al., 2008; Perez and Chokron, 2014), but contrast sensitivity was assessed at only one or a few spatial frequencies. Fortunately, a precise and accurate contrast sensitivity function (CSF) assessment algorithm, called quick CSF (qCSF), was developed within a Bayesian framework (Lesmes et al., 2010). In the current study, the qCSF method is quite suitable to investigate the influence of Bangerter filters on contrast sensitivity over a broad range of spatial frequency and noise levels within a short time. Furthermore, how the degradation of visual acuity and contrast sensitivity correlate with each other at different filter levels could be addressed.

In addition, with the external noise method, scientists developed a perceptual template model (PTM) to characterize the limitations of visual perception (Dosher and Lu, 1998). The PTM explains the changes in visual function by three independent mechanisms. The first factor is internal additive noise, which can amplify or weaken both signal and noise from input stimuli. The second factor is the perceptual template, which determines the capacity to exclude external noise. The third factor is internal multiplicative noise, which behaves according to Weber’s law (Dosher and Lu, 1998). As such, the combination of the PTM and the external noise method is an ideal tool for understanding the mechanisms of visual function loss induced by Bangerter filters.

In summary, the present work aims to evaluate the influence of Bangerter filters with different intensities on visual acuity and contrast sensitivity at a broad range of spatial frequencies and three noise levels. The relationship between visual acuity and contrast sensitivity loss was examined, and the PTM could help us characterize the mechanisms of Bangerter filter–induced visual function defects.

## Method

### Subjects

Ethical approval was obtained from the Ethical Review Committee of Hebei Normal University. Each subject (*n* = 10, ages from 19 to 23 years old) signed an informed consent prior to the experiments. The vision or corrected vision of all subjects was 20/20 or better. No subjects had ophthalmic diseases. This work adhered to the tenets of the Declaration of Helsinki.

### Apparatus and Materials

Bangerter filters (Ryser Optik, St. Gallen, Switzerland) were attached to the surface of subjects’ spectacle lenses. If a subject was emmetropia, he or she would wear plano lenses. Three filter densities were selected: 0.2, 0.4, and 0.8 (from most to least dense). Experimental procedures were programmed and run in MATLAB with the Psychophysics Toolbox (Pelli, 1997). The stimuli of the contrast sensitivity test were presented in a luminance-calibrated cathode ray tube (CRT) monitor (85 Hz, 36.2 cd/m^2^, 1280 × 1024 resolution). During the contrast sensitivity test, subjects viewed the monitor at a distance of 1.71 m in a dim light office. The movement of subjects’ heads was controlled by a chin rest.

### Stimuli

The stimuli for the contrast sensitivity test were vertical gratings and noise images, presented in the center of the display. The spatial frequency of the gratings consisted of ten levels: 0.5, 0.67, 1, 1.33, 2, 2.67, 4, 5.33, 8, and 16 cpd. The diameter of the spatial window was set to 3 cycles of the grating; thus, the size of the spatial window was inversely proportional to the spatial frequency of the grating. The sizes of the noise images and gratings were always identical. Each noise image contained 15 × 15 noise elements (gray squares). In other words, no matter the spatial frequency, each grating cycle contained the same number of noise elements. Each noise image was sampled from a Gaussian distribution with mean 0 and three standard deviations (SD): 0, 0.12, and 0.24, which produced zero-, low-, and high-noise conditions, respectively. It is worth noting that the SD was 0, 12, and 24% of the mean luminance intensity, instead of luminance range. To blur the edge, each grating was covered by a truncated Gaussian envelope, whose SD was 1:6 to the gratings. The visible number of grating cycles was actually less than 3. Besides, the grating did not vary within each noise pixel.

### Procedure

**FIGURE 1:**
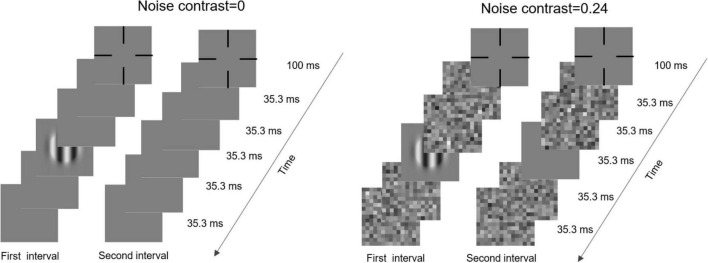
Illustration of a typical trial in zero-noise **(left)** and noisy **(right)** conditions.

The contrast sensitivity was measured by a contrast detection task (Figure 1). In each trial, two intervals were presented and divided by a 500-ms blank. Each interval contained two frames of blank, one frame of blank or grating, and another two frames of blanks and were initialized by a fixation cross, which lasted 100 ms. Each frame lasted 35.3 ms. Under noisy conditions, the two front and back blanks were replaced by noise images. Subjects were instructed to judge which interval included the grating by pressing the buttons of a game controller. A brief beep was presented after each response regardless of whether it was correct.

### Design

The qCSF procedure produced three contrast sensitivity functions, each of which was linked to one external noise level (zero, low, or high). There were 100 trials per noise level, and the trials were randomly presented. Subjects completed a contrast sensitivity test with no filter and with 0.8, 0.4, and 0.2 filters, respectively. Only subjects’ non-dominant eyes were tested, and their dominant eyes were covered by a dark occluder. The hole-in-the-card test was used to assess eye dominance.

#### Perceptual Template Model Analysis

First, a subject’s performance was computed as follows:


(1) $$d^{\prime }=\frac{{\left(\beta c\right)}^{\gamma }}{\sqrt{{\left(A_{f}N_{ext}\right)}^{2\gamma }+A_{m}^{2}N_{mul}^{2}\left({\left(\beta c\right)}^{2r}{\left(A_{f}N_{ext}\right)}^{2\gamma }\right)+{\left(A_{a}N_{add}\right)}^{2}}}$$

where *d*′ represents the performance, *c* denotes the signal contrast, and γ denotes the system’s non-linearity; the internal additive noise and internal multiplicative noise were expressed by *N*_*add*_ and *N*_*mul*_, respectively. *N*_*ext*_ denotes the contrast of external noise and β presents perceptual template gain. To model the Bangerter filter–induced visual function reduction, *A*_*a*_, *A*_*f*_, and *A*_*m*_ were added in front of *N*_*add*_, *N*_*ext*_, and *N*_*mul*_, respectively. In the no-filter condition (baseline), *A*_*a*_, *A*_*f*_, and *A*_*m*_ were all set to 1.0. We found that the slope did not depend on filter levels; thus, the multiplicative noise would be constant (see detailed analysis in Supplementary Material). In other words, *A*_*m*_ was removed from Eq. 1 (Xu et al., 2006; Zhang et al., 2018). In addition, previous studies found that *N_*add*_* and β depended on spatial frequency, but *N_*mul*_* and γ did not (Chen et al., 2014). Finally, we had four models in total: one full model and three reduced models. The full model assumed that both the internal additive noise and the perceptual template changed after subjects wore filters; the reduced Model 1 assumed that only internal additive noise changed after subjects wore filters; the reduced Model 2 assumed that only perceptual template changed after subjects wore filters; the reduced Model 3 assumed that nothing changed after subjects wore filters. The goodness of fit of each model was calculated as follows:


(2) $$r^{2}=1-\frac{\sum {\left(y_{i}-{\hat{y}}_{i}\right)}^{2}}{\sum {\left(y_{i}-\bar{y}\right)}^{2}}$$

where *r*^2^ is the index of goodness of fit; ${\hat{y}}_{i}$ and *y_i_* denote the fitted and original values, respectively; and $\bar{y}$ represents the mean of all original values. The data fitting (PTM) was done on log contrast sensitivity. An *F*-test was used to compare the *r*^2^ values and select the best-fitting model (Huang et al., 2010).

## Results

**FIGURE 2:**
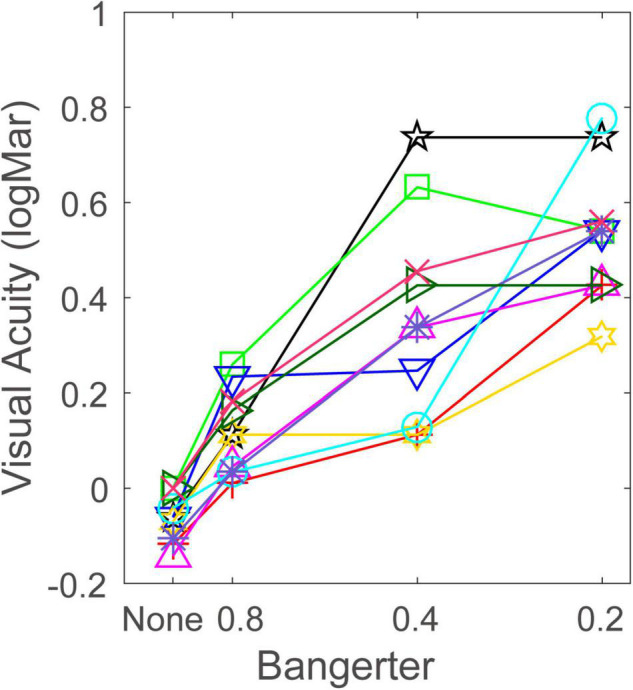
Visual acuity (log MAR) at each Bangerter filter strength. Each symbol and color denotes one subject.

Visual acuity in four viewing conditions is plotted in Figure 2. Visual inspection suggests that the Bangerter filter greatly impaired visual acuity, but this effect also depended on individual differences. The visual acuity when subjects wore none, 0.8, 0.4, and 0.2 filters was –0.059 ± 0.016 (mean ± SE), 0.119 ± 0.028, 0.353 ± 0.068, and 0.529 ± 0.045, respectively. A repeated measurement analysis was conducted on the visual acuity with Bangerter filter intensity as a within-subject factor. The main effect of Bangerter intensity reached significance [*F*(3,27) = 46.618, *p* < 0.001]. Fisher’s least significant difference (LSD) analysis revealed that the none filter produced the best visual acuity, and the subsequent filters resulted in progressive visual acuity degradation (all *p* < 0.05). Paired-samples *t*-tests revealed that the visual acuity with Bangerter filters corresponded to its nominal strength only for the 0.8 and 0.4 filters (all *p* > 0.1). This implied that the variability of visual acuity with Bangerter filters was a major issue. The 0.8, 0.4, and 0.2 filter levels degraded visual acuity by 0.179 ± 0.020, 0.413 ± 0.063, and 0.590 ± 0.044 (mean ± SE, log units) from baseline, respectively. This indicated that Bangerter filters significantly reduced visual acuity, and the amount of reduction highly depended on filter strength. To investigate the effect of individual difference (e.g., refractive error) on the reduction of visual acuity, we performed a Pearson’s Correlation analysis on the relationship between the refractive error and reduction of visual acuity at 0.8, 0.4, and 0.2 filter levels, respectively. We found that the refractive error was negatively correlated with the reduction of visual acuity at 0.8 (*r* = –0.668, *p* = 0.035) and 0.4 filter level (*r* = –0.601, *p* = 0.066). This finding indicated that individual with higher refractive error produced larger reduction by 0.8 and 0.4 filter level. However, the correlation between refractive error and reduction of visual acuity at 0.2 (*r* = 0.240, *p* = 0.504) filter level was not significant (*p* > 0.1). This may account for the increasing visual acuity variation at 0.2 filter level, because nothing was predictable.

**FIGURE 3:**
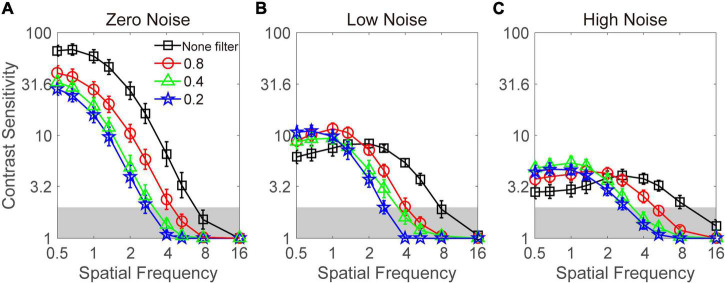
Contrast sensitivity functions under zero- **(A)**, low- **(B)**, and high- **(C)** noise conditions. Dark lines with square symbols, red lines with circle symbols, green lines with triangle symbols, and blue lines with pentagram symbols denote contrast sensitivity with none filter and at the 0.8, 0.4, and 0.2 filter levels, respectively. Data were averaged across subjects. Shaded regions denote contrast sensitivity which is lower than 2. Error bar denotes standard error.

The contrast sensitivity function at three noise levels and four filter levels is plotted in Figure 3. Visual inspection suggests that when external noise was absent, the Bangerter filter degraded the contrast sensitivity at all frequencies; in contrast, the contrast sensitivity was elevated at low frequencies when external noise was present. However, due to floor effect, the measurement of contrast sensitivity at four high spatial frequencies (e.g., >2.67 cpd) was unreliable (<2 log unit). Thus, only the data at six low and intermediate frequencies (≤2.67 cpd) enter into the following statistical analysis. A repeated measurement analysis was conducted on log contrast sensitivity in the zero-noise condition with filter intensity and spatial frequency as within-subject variables. The main effect of filter intensity and spatial frequency and the interaction effect among them all reached significance [*F*(3,27) = 28.467, *p* < 0.001; *F*(5,45) = 239.879, *p* < 0.001; *F*(15,135) = 8.366, *p* < 0.001]. LSD revealed that: (1) 0.8, 0.2, and 0.1 filters resulted in lower contrast sensitivity than none filter at all spatial frequencies (all *p* < 0.05); (2) 0.8 filter resulted in lower contrast sensitivity than 0.2 filter at all spatial frequencies (all *p* < 0.05); (3) 0.8 filter resulted in lower contrast sensitivity than 0.4 filter at 0.5, 0.67, 1, and 1.33 cpd (all *p* > 0.05), instead of 2 and 2.67 cpd (all *p* < 0.05); and (4) the contrast sensitivity between 0.4 and 0.2 filters was comparable at all spatial frequencies (all *p* > 0.1). In summary, when external noise was absent, Bangerter filters greatly degraded the contrast sensitivity. Furthermore, the more intensive the filter was, the worse the contrast sensitivity became.

For contrast sensitivity when low noise was present, a repeated-measures analysis was conducted on log contrast sensitivity with filter intensity and spatial frequency as within-subject variables. The main effect of spatial frequency and the interactions between filter intensity and spatial frequency reached significance [*F*(5,45) = 21.990, *p* < 0.001; *F*(15,135) = 14.594, *p* < 0.001]. But the main effect of filter intensity failed to reach significance [*F*(3,27) = 1.919, *p* = 0.150]. LSD revealed that: (1) the contrast sensitivity with none filter was significantly (or marginally) lower than that with 0.8 filters at 0.5, 0.67, 1, and 1.33 cpd (all *p* < 0.084), but higher than 0.8 filter at 2.67 cpd (*p* = 0.008); (2) the contrast sensitivity with none filter was significantly (or marginally) lower than that with 0.4 filter at 0.5 cpd (*p* = 0.061), but higher than that with 0.4 filter at 2 and 2.67 cpd (all *p* < 0.05); (3) the contrast sensitivity with none filter was significantly lower than that with 0.2 filter at 0.5 and 0.67 cpd (all *p* < 0.05), but higher than that with 0.2 filter at 2 and 2.67 cpd (all *p* < 0.05); (4) the contrast sensitivity with 0.8 filter was significantly (or marginally) higher than that with 0.4 filter at 1.33, 2, and 2.67 cpd (all *p* < 0.059); and (5) the contrast sensitivity with 0.4 filter was significantly higher than that with 0.2 filter at 2.67 cpd (*p* = 0.019). Other comparisons between different filter levels were negative (all *p* > 0.1). These results suggested that when low noise was present, a crossover effect was observed.

For contrast sensitivity when high noise was present, a repeated-measures analysis was conducted on log contrast sensitivity with filter intensity and spatial frequency as within-subject variables. The main effect of spatial frequency and the interaction effect among filter intensity and spatial frequency reached significance [*F*(5,45) = 5.387, *p* < 0.001; *F*(15,135) = 9.516, *p* < 0.001]. However, the main effect of filter intensity failed to reach significance [*F*(3,27) = 1.650, *p* = 0.201]. LSD revealed that: (1) the contrast sensitivity with none filter was significantly (or marginally) lower than that with 0.4 (*p* = 0.006) and 0.2 (*p* < 0.001) filters at 0.5 cpd, that with 0.8 (*p* = 0.077), 0.4 (*p* = 0.006), and 0.2 (*p* < 0.001) filters at 0.67 cpd, that with 0.8 (*p* = 0.071), 0.4 (*p* = 0.004), and 0.2 (*p* < 0.001) filters at 1 cpd, and that with 0.4 (*p* = 0.020) at 1.33 cpd; (2) the contrast sensitivity with 0.8 filter was significantly (or marginally) lower than that with 0.4 filter at 1 cpd (*p* = 0.093), but higher than that with 0.4 filter at 2.67 (*p* = 0.094); (3) the contrast sensitivity with 0.8 filter was significantly higher than that with 0.2 filter at 2 and 2.67 cpd (all *p* < 0.05); and (4) the contrast sensitivity with 0.4 filter was significantly (or marginally) higher than that with 0.2 filter at 1.3 (*p* = 0.077) and 2 (*p* = 0.034) cpd. Other comparisons between different filter levels were not significant (all *p* > 0.1). These results suggested that when high noise was present, a crossover effect was also present.

To explore whether the influence of Bangerter filters on visual acuity and contrast sensitivity is identical, we computed the reduction of two types of visual function at three filter levels. Because visual acuity was measured in a noise-free environment, only contrast sensitivity at the zero-noise level was entered into the correlation analysis. At first, a Pearson correlation analysis was performed on the relationship between the reduction of visual acuity and contrast sensitivity at each spatial frequency. No significance was detected (all *p* > 0.1). Then, we computed the area under the log contrast sensitivity function (AULCSF, in log_10_ units), which served as the index of contrast sensitivity across all spatial frequencies (Koop et al., 1996; Zhang et al., 2018; Wu et al., 2020, 2021). Pearson correlation analysis revealed that the magnitude of the visual acuity reduction did not correlate with that of AULCSF reduction at each filter level (all *p* > 0.1). These findings suggested that the reduction of visual acuity with a filter could not predict that of contrast sensitivity.

**FIGURE 4:**
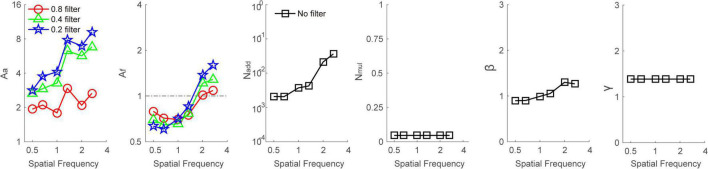
*A*_*a*_, *A*_*f*_, *N*_*add*_, *N*_*mul*_, β, and γ as a function of spatial frequencies at baseline (dark square), 0.8 (red circle), 0.4 (green triangle), and 0.2 (blue pentagon) filter levels, respectively. Different viewing conditions shared *N*_*add*_, *N*_*mul*_, β, and γ.

To illustrate the mechanisms of Bangerter filter–induced loss of contrast sensitivity, we first averaged the data across subjects and then fitted them with the PTM. Because the contrast sensitivity at four high spatial frequencies could not be observed due to the high intensity of Bangerter filters (e.g., 0.2 filter), only the data at the other six frequencies entered into the following analysis. We selected the model that had the fewest parameters while producing an *r*^2^ comparable to that of the full model. The *r*^2^ values of the full model, reduced Model 1, reduced Model 2, and reduced Model 3 were 91.8, 77.0, 54.0, and 49.1%, respectively. The *F*-test revealed that the *r*^2^ of the full model was significantly higher than that of any reduced models (all *p* < 0.001). Thus, the full model was selected as the best-fitting model. The best-fitted parameters are plotted in Figure 4. Averaged across all spatial frequencies, *A*_*a*_ was 2.253 ± 0.184, 4.595 ± 0.755, and 5.765 ± 1.042 at 0.8, 0.4, and 0.2 filter levels, respectively. This finding indicated that the higher intensity of the filter resulted in higher internal additive noise. In contrast, *A*_*f*_ seems to be lower than 1 at low spatial frequencies (≤1.33 cpd) but higher than 1 at intermediate spatial frequencies (>1.33 cpd). Average across low spatial frequencies, *A*_*f*_ was 0.739 ± 0.021, 0.691 ± 0.027, and 0.700 ± 0.055 at 0.8, 0.4, and 0.2 filter levels, respectively. In contrast, on average across intermediate spatial frequencies, *A*_*f*_ was 1.051 ± 0.036, 1.250 ± 0.037, and 1.484 ± 0.109 at the 0.8, 0.4, and 0.2 filter levels, respectively. This indicated that the perceptual template was impaired at intermediate spatial frequencies but improved at low spatial frequencies by Bangerter filters. In addition, the changes in the perceptual template were also dependent on filter levels.

## Discussion

In the current study, we examined the effect of Bangerter filters with different intensities on visual acuity and contrast sensitivity function. We found that both functions were degraded by Bangerter filters, but the visual acuity degradation was inconsistent with the manufacturer’s specification or correlated with the contrast sensitivity reduction. In addition, when external noise was present, contrast sensitivity was decreased at intermediate to high spatial frequencies but elevated at low spatial frequencies. Furthermore, PTM analysis revealed that measurement of internal additive noise and perceptual templates were biased by Bangerter filters.

An important consideration in amblyopia treatment is whether the acuity degradation after the use of a Bangerter filter is consistent with the manufacturer’s specifications. However, visual acuity at the 0.2 filter level was significantly higher than 0.2. Additionally, the individual difference in visual acuity grew as the filter intensity increased. Researchers found a characteristic pattern of microbubbles in Bangerter filters (Pérez et al., 2010). The strength of a Bangerter filter is related to the number of microbubbles in it. That is, the more microbubbles a filter contains, the more serious the image degradation it induces. If subjects learn to view scenes through a clearer portion of the Bangerter filter, better visual acuity will be observed. In the current study, subjects were asked to view an E chart or monitor through the center, and the same piece of filter material was used for each subject. However, we still could not ignore the individual difference in Bangerter filters response. That is to say, individual visual system may adapt to Bangerter filter in different degree. Overall, poor correspondence between the labeled density designation and the actual density of Bangerter filters complicates the work of clinicians. Thus, developed customized filter material which could perfectly match patients’ demand is very necessary.

Exploring the influence of Bangerter filters on the contrast sensitivity function is another key point of the current study. This issue has been investigated by previous studies, but our experimental design still has significant innovations. Due to the limitation of traditional CSF assessment, some researchers measured only one spatial frequency without changing any noise levels (e.g., Odell et al., 2008; Perez and Chokron, 2014). We found that contrast sensitivity was degraded by filters at all spatial frequency conditions, and the degree of reduction was dependent on the filter level.

The filter–induced degradation in visual acuity and contrast sensitivity did not correlate with each other. This suggested that visual acuity and contrast sensitivity were two distinctive visual functions. One uses high-contrast stimuli, while the other uses low-contrast stimuli. It has been reported that dichoptic CSF may be considered to measure visual performance in patients with altered binocular vision (Barboni et al., 2020). In the context of amblyopia treatment, whether to balance the visual acuity or the contrast sensitivity of the two eyes is an interesting question.

In the current study, the floor effect was not friendly to the measurement of contrast sensitivity at high spatial frequencies with high intensity of filters. Because the contrast detection task at those conditions was too hard, subjects might not perceive any gratings, even when the gratings were present in 100% contrast. This may be a problem for the statistical analysis, because it can artificially induce significant interactions between spatial frequency and filter level. We only focus on the pattern of data at low and intermediate spatial frequencies (e.g., <4 cpd), and our main conclusion is robust. That is, when external noise was absent, the more intensive the filter was, the worse the contrast sensitivity became; and Bangerter filters produced a cross effect when low or high noise was present.

The dependency of filter effects on external noise levels is another key point of the current work. First, when external noise was presented, the pattern of the contrast sensitivity function was totally different from that when external noise was absent. Specifically, the contrast sensitivity at low spatial frequencies was improved instead of degraded. We speculate that Bangerter filters suppress the energy of noise more than that of the signal grating at low spatial frequencies. This account is inspired by the findings from previous studies, in which luminance attenuation can improve subjects’ performance in a motion direction discrimination task at low temporal frequencies, instead of high temporal frequencies (Allard and Arleo, 2017a,b). They assumed that the visibility of irrelevant masking information at high temporal frequencies, which disturb the motion sensitivity, was reduced by neutral density filters. An analogous phenomenon has been observed in face recognition studies. For example, blur can filter out salient edges of the coarsely sampled picture, which is harmful to the performance of face recognition because only the information at lower spatial frequencies is useful. Thus, spatial blur can promote face recognition. In our study, each external noise image contain 15 × 15 elements, which is at high spatial frequency. Thus, Bangerter filters may greatly reduce the visibility of irrelevant external noise at high spatial frequencies and improve our ability of perceiving other relevant information (e.g., grating detection at lower frequencies). With the help of PTM, we found that the measurements of internal additive noise and perceptual template were biased after subjects wore Bangerter filters. This is because both internal additive noise and perceptual template belonged to internal process, which should not be influenced by any filters outside. We guess that Bangerter filters may attenuate the contrast of input signal and change the intensity of external noise (reduce it at low spatial frequencies but amplify it at high spatial frequencies). These findings also indicated a limitation of the use of the PTM. In real life, individuals must frequently detect targets against a noisy background, e.g., identify pedestrians in foggy weather. These findings also lead us to ponder whether it is proper to simulate visual defects in several populations, e.g., people with cataracts and those with vitreous haze in uveitis, using Bangerter filters.

## Data Availability Statement

The raw data supporting the conclusions of this article will be made available by the authors, without undue reservation.

## Ethics Statement

The studies involving human participants were reviewed and approved by Ethical Review Committee of Hebei Normal University. The patients/participants provided their written informed consent to participate in this study.

## Author Contributions

PZ collected and analyzed the data. PZ, YZ, and DW designed the experiment and wrote the manuscript. All authors revised the manuscript, contributed to the article, and approved the submitted version.

## Conflict of Interest

The authors declare that the research was conducted in the absence of any commercial or financial relationships that could be construed as a potential conflict of interest.

## Publisher’s Note

All claims expressed in this article are solely those of the authors and do not necessarily represent those of their affiliated organizations, or those of the publisher, the editors and the reviewers. Any product that may be evaluated in this article, or claim that may be made by its manufacturer, is not guaranteed or endorsed by the publisher.
