# Supplementary material for: The Effect of Bangerter Filters on Visual Acuity and Contrast Sensitivity With External Noise
Source: Front Neurosci. 2022 May 13;16:804576. doi: 10.3389/fnins.2022.804576 (PMC9136063; doi:10.3389/fnins.2022.804576)
Supplement: Supplementary file 1 [file Table_1.DOCX]

**Supplementary Information**

**Slope Check in Measurement of the Contrast Sensitivity Function**

At first, the performance of an observer was defined by the following equation:

$P_{i,j}\left( x \right)=\gamma+\left( 1-\gamma-\lambda\right)\left( 1-exp\left( -{10}^{s\left( {log}_{10}\left( x \right)-{log}_{10}\left( \tau_{i,j} \right) \right)} \right) \right)$, Eq. S1

where *P* denotes the percent correct. *i* and *j* mean the spatial frequency and external noise condition. Guessing rate (*γ*) and lapse rate (*λ*) is set to 0.5 and 0.02, respectively. *τ* is the contrast threshold at 80.3% correct performance level. *s* denotes the slope of psychometric function. Then, *s* was estimated by a maximum-likelihood procedure. A repeated measurement analysis was performed on the slopes with viewing condition (baseline, 0.8, 0.4, and 0.2, respectively) as a within-subject variable. We found that the slopes in different filter levels were comparable (F(3, 27) = 0.108, *p* = 0.954).

**Fitting the Perceptual Template Model (PTM)**

The raw threshold over spatial frequencies could derived one psychometric functions with six contrast level, corresponding to 60%, 70%, 78%, 84%, 90%, and 99% correct. Because the threshold at four high frequencies could not be measured with bangerter filters, only the data at other six spatial frequencies entered into the following analysis. Six spatial frequencies, three external noise levels, and four filter levels produced 72 psychometric functions. Thus, a total of 432 data points were included in model fitting
